# Receptor activity-modifying protein dependent and independent activation mechanisms in the coupling of calcitonin gene-related peptide and adrenomedullin receptors to Gs

**DOI:** 10.1016/j.bcp.2017.07.005

**Published:** 2017-10-15

**Authors:** Michael J. Woolley, Christopher A. Reynolds, John Simms, Christopher S. Walker, Juan Carlos Mobarec, Michael L. Garelja, Alex C. Conner, David R. Poyner, Debbie L. Hay

**Affiliations:** a Institute of Clinical Sciences, University of Birmingham, Edgbaston, Birmingham, UK; b School of Biological Sciences, University of Essex, Wivenhoe Park, Colchester CO4 3SQ, UK; c School of Life and Health Sciences, Aston University, Aston Triangle, Birmingham, UK; d School of Biological Sciences, University of Auckland, Auckland, New Zealand

**Keywords:** Adrenomedullin (PubChem CID: 56841671), Calcitonin gene-related peptide (PubChem CID: 56841902), cAMP (PubChem CID: 6076), Adrenomedullin, Calcitonin gene-related peptide, GPCR, Receptor activity-modifying protein, Molecular modeling, Molecular dynamics

## Abstract

Calcitonin gene-related peptide (CGRP) or adrenomedullin (AM) receptors are heteromers of the calcitonin receptor-like receptor (CLR), a class B G protein-coupled receptor, and one of three receptor activity-modifying proteins (RAMPs). How CGRP and AM activate CLR and how this process is modulated by RAMPs is unclear. We have defined how CGRP and AM induce Gs-coupling in CLR-RAMP heteromers by measuring the effect of targeted mutagenesis in the CLR transmembrane domain on cAMP production, modeling the active state conformations of CGRP and AM receptors in complex with the Gs C-terminus and conducting molecular dynamics simulations in an explicitly hydrated lipidic bilayer. The largest effects on receptor signaling were seen with H295A^5.40b^, I298A^5.43b^, L302A^5.47b^, N305A^5.50b^, L345A^6.49b^ and E348A^6.52b^, F349A^6.53b^ and H374A^7.47b^ (class B numbering in superscript). Many of these residues are likely to form part of a group in close proximity to the peptide binding site and link to a network of hydrophilic and hydrophobic residues, which undergo rearrangements to facilitate Gs binding. Residues closer to the extracellular loops displayed more pronounced RAMP or ligand-dependent effects. Mutation of H374^7.47b^ to alanine increased AM potency 100-fold in the CGRP receptor. The molecular dynamics simulation showed that TM5 and TM6 pivoted around TM3. The data suggest that hydrophobic interactions are more important for CLR activation than other class B GPCRs, providing new insights into the mechanisms of activation of this class of receptor. Furthermore the data may aid in the understanding of how RAMPs modulate the signaling of other class B GPCRs.

## Graphical abstract

**Figure:**
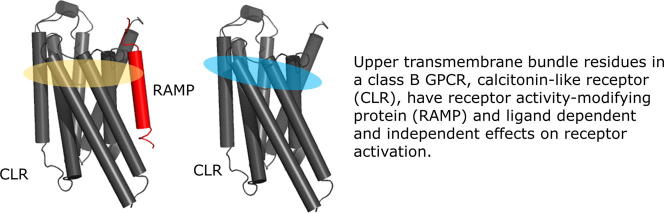

## 1 Introduction

Class B G protein-coupled receptors (GPCRs) are a small but physiologically and therapeutically important sub-group of the GPCR superfamily. They are involved in a diverse range of physiological responses such as vasodilation, stress, digestion and glucose homeostasis [2]. This has made them important drug targets for a range of human diseases including diabetes, obesity, cancer, cardiovascular disease and migraine [22].

Calcitonin gene-related peptide (CGRP) and adrenomedullin (AM) are peptide ligands that bind to heterodimers of the class B calcitonin receptor-like receptor (CLR), in association with a single transmembrane receptor activity-modifying protein (RAMP1, 2 or 3). CGRP binds the CLR/RAMP1 complex with high affinity but AM can also bind to this receptor. AM binds to both CLR/RAMP2 (AM_1_ receptor) and CLR/RAMP3 (AM_2_ receptor) with high affinity. At the human AM receptors, CGRP binds very weakly [1], [5], [52]. CGRP and AM are potent vasodilators and have been implicated in cardiovascular disease [55]. The CGRP system is under intense drug scrutiny as a target for migraine [40]. Thus, understanding the activation mechanism of these receptors and how RAMPs affect this, is of considerable importance for drug development.

GPCR activation, once thought to be a simple on/off switch mechanism, has become increasingly defined by its complexity. A receptor is able to exist in multiple conformations, stabilized by different ligands, resulting in activation or inactivation of several possible signaling pathways and requiring the allosteric effects of a bound G protein to achieve maximum affinity for agonist binding [16], [28], [29], [47].

Most understanding of the activation mechanism of GPCRs comes from class A GPCRs, where activation involves conformational changes in the transmembrane helices (TM). The movement of the extracellular ends of TM helices is a key process in the activation mechanism of GPCRs, as is the stabilization of conformational changes through inter-helical interactions of both polar and hydrophobic residues, often involving conserved motifs [18], [47]. Individual agonists produce a variety of changes around their binding pockets, but these converge to produce changes in the upper half of the TM bundle, which are propagated to the cytoplasmic end of the bundle. The most significant movement is a rigid-body rotation of the bent TM6 (accompanied by some torsional changes in the vicinity of P5.50), thus opening the cleft required for G protein-binding [45], [47].

Three published X-ray crystal structures of class B GPCRs, all in the inactive conformation [23], [26], [43], were available when this article was submitted; more recent cryo-electron microscopy structures of the active form are discussed below. These three class B X-ray structures show structural similarity with the class A crystal structures on the intracellular (G protein-binding) half of the TM bundle. The extracellular side however is more open. Despite this, molecular dynamics simulations of the corticotropin-releasing factor receptor 1 (CRF1R) suggested that activation of the receptor involved an outward movement of TM5 and 6, consistent with class A [42]. However, in the inactive class B X-ray structures TM6 is not uniformly bent as in class A; TM6 is relatively straight in the glucagon receptor [43], [26]. Consequently, the anticipated outward movement of TM6 in class B GPCRs may have a more significant torsional component. A network of hydrophilic interactions between TM helices has been suggested in the class B glucagon-like peptide-1 receptor (GLP-1R) [58], [60]. Conserved polar residues within the TM helices of the GLP-1R are important in coordinating either global receptor activation conformational changes or for fine-tuning responses, leading to biased signaling. The role of hydrophobic residues within the TM bundle in class B GPCRs largely remains to be addressed, but in class A GPCRs hydrophobic residues play an important role in facilitating the conformational change [18], [47].

RAMPs can significantly alter the pharmacology and signaling of class B GPCRs but the mechanisms are poorly understood [11], [21], [35], [48], [53], [54], [57]. For CGRP and AM receptors, there is a direct interaction of the RAMP extracellular domains with the C-terminus of the peptide [10], but there is also evidence for RAMP affecting the GPCR extracellular loops (ECLs) [51]. Thus their effects on the entire GPCR need to be considered.

How RAMPs affect the activation mechanisms of class B GPCRs is not known. Furthermore, it is unclear whether CLR has unique features compared to other class B GPCRs, given its obligate requirement for RAMPs. We have addressed these questions in CGRP and AM receptors using an integrated experimental and computational approach, to provide a model with which to compare the effect of different RAMPs on the GPCR activation mechanism. We used structural models to select amino acids that we hypothesized are most likely to be involved in stabilizing conformational changes. Mutants were pharmacologically characterized and computational simulation of the inactive to activation transition of CLR/RAMP complexes with Gs was used to interpret the results. This has allowed us to suggest a mechanism for receptor activation leading to Gs coupling for CLR. This shows commonalities but also special features compared to other class B GPCRs.

## 2 Materials and methods

### 2.1 Materials

Human αCGRP and human AM (AM 1–52), were from American Peptide (Sunnyvale, CA, USA) or Bachem (Bubendorf, Switzerland). Forskolin was from Tocris Bioscience (Bristol, UK). LANCE cAMP assay kits and all reagents and plates were from PerkinElmer (Waltham, MA, USA). [^125^I]-human alpha iodohistidyl^10^-CGRP and [^125^I]-human (13–52) iodotyrosyl^52^adrenomedullin (^125^I-AM) were also purchased from Perkin Elmer. All other chemicals were from Sigma.

### 2.2 Expression constructs and mutagenesis

Human CLR with an N-terminal haemagglutinin (HA) epitope was mutated using a method based on the Quik Change II site-directed mutagenesis kit (Stratagene, Cambridge, UK) and described previously [4]. Human RAMP 1 with an N-terminal myc epitope tag [56], human RAMP2 with an N-terminal FLAG epitope tag [38] and untagged human RAMP3 were also used [12].

### 2.3 Cell culture and transfection

Culture of Cos7 cells was performed as previously described [3]. These cells were originally obtained from the American Type Culture Collection and cells were used between passages 16 and 32. Cells were cultured in Dulbecco’s Modified Eagle Medium supplemented with 8% heat-inactivated foetal bovine serum and kept in a 37 °C humidified 95% air, 5% CO_2_ incubator. For cAMP assays and cell surface expression ELISAs, cells were seeded into 96-well plates at a density of 15,000 cells per well (determined using a Countess Counter™, Invitrogen, Carlsbad, CA, USA) 1 day before transfection. Cells were transiently transfected using polyethylenimine (PEI) as described previously [3] using a 1:1 ratio of CLR to RAMP.

### 2.4 Cell surface expression ELISA

Cell surface expression of all RAMP/HA-CLR receptor complexes was assessed by measuring HA-CLR expression in an ELISA as previously described [12], [4], with some modifications. Paraformaldehyde (8%, 100 µL) in PBS was added to each well of a 96 well plate containing transfected cells and the plate was incubated at room temperature with gentle shaking for 20 min. The cells were washed twice in PBS (100 µL per well). A 1% solution of BSA or 10% goat serum (100 µL) in PBS was added to each well to block nonspecific protein interactions and incubated at room temperature for 1 h. The wells were aspirated and 50 µL of anti-HA monoclonal primary antibody (Sigma H-9658), diluted 1:2000 in 1% BSA or 1% goat serum in PBS, was added to each well and incubated at room temperature for 1 h. The wells were aspirated and washed once in PBS before adding 50 µL anti-mouse horseradish peroxidase-conjugated secondary antibody (Sigma A-4416), diluted 1:2000 in 1% BSA or 1% goat serum in PBS at room temperature for 1 h. The wells were aspirated and washed twice in PBS before adding 50 µL of o-phenylenediaminedihydrochloride (OPD) solution and incubating this in the dark for 15 min. H_2_SO_4_ (50 µL, 0.5 M) was added to stop the reaction and absorbances were read at 490 and 650 nm. The wells were aspirated and washed twice in PBS. Cresyl violet working solution (50 µL) was added to each well and incubated at room temperature for 30 min. The wells were washed once in PBS and 1% sodium dodecyl sulphate was added and incubated at room temperature with gentle shaking for 10 min. The absorbance at 595 nm was measured and a ratio (A490-A650)/A595) calculated for each well. For selected mutants, a myc antibody (Millipore, Billerica, MA, U.S.A. OP10, diluted 1:250) or FLAG antibody (Sigma-Aldrich, St. Louis, MO, U.S.A. M2 F1804, diluted 1:1000) was used to also quantify RAMP1 or RAMP2 expression, respectively.

### 2.5 cAMP assay

Transfected cells were stimulated with agonist and lysates prepared for cAMP assay, essentially as previously described [52]. However, this protocol was modified for a LANCE cAMP assay (Perkin Elmer, Waltham, MA, USA) [25]. Briefly, on the day of the assay, cells were serum-deprived in DMEM containing 1 mM isobutyl methyl xanthine and 0.1% BSA for 30 min. Peptides, reconstituted to 1 mM in ultra-pure water, were diluted in the same medium to give a final concentration range of 1 pM to 1 μM. These concentrations were selected, based on the known potencies of the peptides at the receptors. Peptides were added to cells and incubated at 37 °C for 15 min. The contents of the wells were then aspirated, and 50 μL of ice-cold absolute ethanol was added and allowed to evaporate. cAMP was extracted by adding 50 μL of LANCE detection buffer (50 mM HEPES (pH 7.4), 10 mM CaCl_2_ and 0.35% Triton X-100). The plates were gently shaken at room temperature for 15 min. Five μL of each cell lysate was transferred to a 384 well white opti-plate (Perkin Elmer, Waltham, MA, USA), followed by 5 μL of cAMP antibody diluted in detection buffer. The plate was sealed and incubated in the dark for 30 min at room temperature before adding 10 μL of the detection mix to all wells. These latter parts were performed by hand or by using a Perkin Elmer Janus automated workstation. The plate was incubated in the dark for 1 or 4 h before reading using an Envision plate reader (PerkinElmer, Waltham, MA, USA). The quantity of cAMP produced was determined from the raw data using a cAMP standard curve, included in each assay.

### 2.6 Radioligand binding

This was performed on membranes made from transfected Cos7 cells, essentially as described elsewhere [12]. The membranes were resuspended in 2 mM MgCl_2_, 0.5% BSA and incubated for 30 min, at room temperature, with 10 pM radioligand and increasing concentrations of unlabelled CGRP or AM, as appropriate. Non-specific binding was defined using 1 μM CGRP or AM. Incubations were terminated by centrifugation.

### 2.7 Data analysis for cell surface expression ELISA

For each individual transfection, representing an individual experiment, the mean (A490-A650)/A595 value for vector alone or vector/RAMP was subtracted from the mean (A490-A650)/A595 value for wild-type (WT) or mutant experimental replicates, giving values that were corrected for background. Given the day-to-day variation in these values due to transient transfection and other factors such as reagent temperature, the data were expressed as a percentage of WT for each experiment to allow the data to be combined. Statistical significance between WT and mutants was then determined using one-way ANOVA, followed by a post hoc Dunnett’s test. For all assays, significance was accepted at p < 0.05. ANOVA was used because all mutants were assayed together. The number of individual experiments is indicated in the tables.

### 2.8 Data analysis for cAMP assay and radioligand binding assays

Data analysis was performed in GraphPad Prism 6 or 7 (GraphPad Software Inc., San Diego, CA, USA). cAMP values were interpolated from the raw data using the cAMP standard curve. Data were fitted to obtain concentration–response curves using a three parameter logistic equation. From these curves, basal, pEC_50_ and E_max_ values were obtained. pEC_50_ and E_max_ values are presented as the mean ± SEM of values from individual data sets, which each had three technical replicates. The data were tested for statistical significance versus WT using an unpaired *t*-test because the experimental design used a WT receptor on each 96 well plate together with three mutants that were randomized between plates. Hence this is effectively a paired experimental design. Curves are presented as the combined means of data, with the number of individual experiments indicated in the tables and significance was accepted at p < 0.05. Blinding was not used during analysis.

To further compare effects of mutations, the differences in relative activity (RA) between the WT and mutant receptors were considered [30]. The Log(RA) for each mutant and corresponding WT were calculated as log[mutantE_max_/mutantEC_50_] and log[WTE_max_/WTEC_50_]; this was corrected for cell surface expression of CLR using the ELISA data, by dividing by the expression relative to WT. The 95% confidence limits for each Δlog(RA) value were computed to identify values different from 0; errors from both curve fitting and cell surface expression were propagated during the process (http://www.met.rdg.ac.uk/~swrhgnrj/combining_errors.pdf). Differences between Δlog(RA) were investigated by one-way ANOVA followed by Tukey’s multiple comparison test to compare individual means between RAMPs.

Radioligand binding data were analysed in Graphpad Prism as described for the cAMP assays. Since the radioligand was used at a concentration below its Kd, changes in affinity will be reflected most sensitively by a decrease in the amount of specific binding relative to WT (as it is easier to identify a 50% reduction in the latter than a 2-fold decrease in pIC_50_). Thus for each mutant, the total specific binding relative to WT was calculated from the span values and the 95% confidence limits were calculated. This relies on the B_max_ value not changing; while this is consistent with our ELISA data, we cannot rule out a contribution to Kd arising from a small change in receptor expression.

### 2.9 Molecular dynamics simulations

Molecular dynamics simulations have been run on three systems: the TM domain of the inactive receptor alone (CLR helices in red in the Figs.), the active to inactive transition (CLR helices in purple in the Figs.), again for the TM domain of the receptor in the absence of ligand and RAMP and for the full active CLR (I32-K402) in the presence of RAMP1 (C27-V148) and CGRP peptide (CLR helices individually colored in Figs.). Together these models give an indication of the orientation of the helices in the active and inactive states and in the absence and presence of the peptide ligand.

#### 2.9.1 TM domain models of inactive CLR

Three inactive TM domain models of CLR were prepared. WT, mutant V190A, and mutant H374A. These models were built with a multiple-template modeling strategy [34] which used only class B structural templates. One hundred starting models were built using Modeller [41], and the top scored models ranked with the DOPE scoring function [19] were visually inspected to select the best model using expert Modeller criteria. The dynamic properties of the models were assessed using all-atom molecular dynamics simulations. Briefly, the receptors were immersed in a 1-palmitoyl-2-oleoyl-sn-glycero-3-phosphocholine (POPC) bilayer and hydrated with the TIP3P water model, ions were added up to a 0.15 M concentration. The simulation was run with ACEMD [20] at 300 K, with the Amber ff14SB [24] forcefield for the protein and lipid14 [17] for the lipids. The production runs were 120 ns for each TMD model, totaling 360 ns.

#### 2.9.2 The inactive to active transition of CLR

Homology models of the transmembrane and loop regions of the inactive CLR were generated using Modeller [41] utilizing the X-ray crystallographic coordinates of the glucagon receptor and CRF1R [43], [23]. One thousand models were generated which were subsequently refined and ranked using the membrane relax module of Rosetta [7]. Each of the original models was refined 3 times generating 3000 structures in total. The active CLR model was generated in a manner similar to the inactive structure, except the cytoplasmic half of TM6 (residues 318–338) was allowed to freely rotate and translate. In addition, four reference points between TMs 3 and 6 were used to limit the conformational freedom of TM6. Prior to Rosetta refinement, a G_αs_ fragment was inserted into the cavity between TMs 3, 5, 6 and 7 using Modeller. Initial tests using the adenosine A_2A_ receptor and β_2_ adrenergic receptor suggested that this method could reliably predict the orientation of the active TM6 position based on an inactive starting structure (data not shown). The best scoring active and inactive models were used as starting conformations for essential dynamics simulation [33]. Each protein was embedded in an equilibrated solvated membrane consisting of 280 POPC lipids. NaCl was added at a concentration of 150 mM, with extra Cl^-^ ions added to the solvent to neutralize the system. Protonation states of charged residues were determined using ProPka [9] prior to the simulation start. The simulations (100 ns) were performed in triplicate using Gromacs [49] at 310 K for both the active and inactive receptor states using a random number for the initial seed.

A trajectory consisting of the concatenated active and inactive conformations which included the TMs and the loops was built and used for principal component analysis. The subsequent covariance matrix of positional fluctuations was built and diagonalized. A single eigenvector with a non-zero eigenvalue resulted from the analysis and was used as a reaction coordinate for an essential dynamics simulation [33]. During the essential dynamics simulation the distance along the first eigenvector was increased in fixed increments per step to drive the system from the inactive to the active state. This was also done from the active to inactive conformation. Simulations were performed on the 30 ns timescale with fixed increments of 1.2 × 10^−6^ nm per each simulation step (2 fs). Each simulation was performed 10 times. The simulations were then combined using the best scoring snapshot, using the Rosetta scoring function at each timestep, and parsed such that the TM region alone was visually inspected.

#### 2.9.3 The full active model of CLR with RAMP1 and Gs fragment

A full active model of CLR (I32-K403) in the presence of RAMP1 (C27-V148), CGRP and the C-terminal tail of G_αs_ (R374-L394) [39] was also generated, as described in [54], but with the 5EE7 glucagon structure also used as a template [26]; two 500 ns simulations were run as for the three inactive models (2.9.1). Two 500 ns simulations of the corresponding CLR/RAMP2/AM/Gs C-terminal tail model were also run. The four MD trajectories are available from the Essex Research Data Repository, doi: https://dx.doi.org/10.5526/ERDR-00000066.

## 3 Results

### 3.1 Identification and selection of residues for alanine substitution

**Fig. 1:**
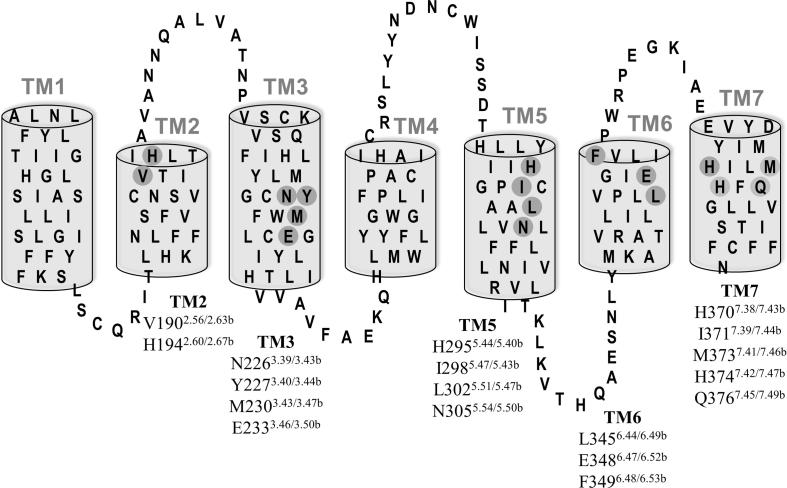
Snake plot of TM residues of CLR; residues selected for alanine substitution are shaded in grey. These residues are numbered according to their primary sequence, followed by the class A/class B numbering in superscript (e.g. V190^2.56/2.63b^).

The aim of this study was to investigate CLR residues situated within the TM helices that may affect conformation through intramolecular interactions; these were studied in a heteromer with RAMP1, RAMP2 or RAMP3. Using previously described models [51], [56], residues were largely chosen to reside below the predicted peptide binding site, to reside close to each other within the TM core and to have a predicted inward or helix facing orientation, so that they were likely to have potential effects on conformational changes occurring on activation. Consequently, 18 residues from TM 2, 3, 5, 6 and 7 were selected (Fig. 1). These residues are numbered according to their primary sequence, and in Fig. 1 this is followed by the class A/class B numbering in superscript (e.g. V190^2.56/2.63b^); the class A numbering scheme is based on that of Ballasteros and Weinstein and the class B numbering scheme is based on that of Wootten et al. [60], [6]. This nomenclature is used in Fig. 1 to enable comparisons to other studies. In the text, tables (apart from Table 5) and other figures we use mainly the primary sequence position.

### 3.2 Effect of alanine substitution on receptor cell surface expression

**Fig. 2:**
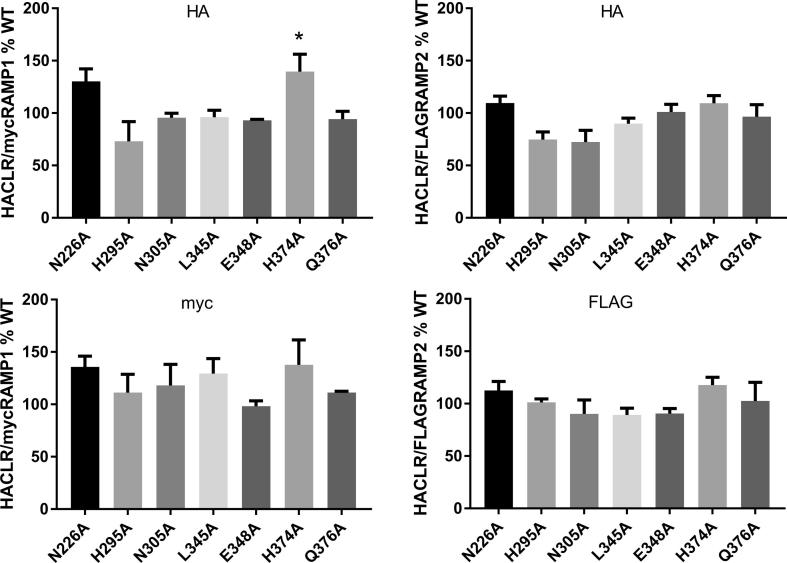
Cell surface expression data for selected mutants showing CLR (HA) and RAMP1 (myc) or RAMP2 (FLAG) expression. Each bar is the combined mean ± s.e.m. from 3 to 6 independent experiments, each performed with triplicates or quadruplicates.

There was no statistically significant difference in the cell surface expression of the CLR alanine substitutions compared to WT CLR with the exception of H374A (with RAMP1), which had increased expression (Table 1, Fig. 2). For a selection of mutants, including H374A we also assessed RAMP cell surface expression and found no significant differences compared to WT (Fig. 2).

**Table 1:** Cell surface expression (C.S.E.) and cAMP production for RAMP1/CLR mutants, using CGRP or AM as agonist.

Mutant	C.S.E.	n	CGRP						AM					
			pEC_50_		E_max_ cAMP (nM)		ΔLog(RA)	n	pEC_50_		E_max_ cAMP (nM)		ΔLog(RA)	n
			WT	Mutant	WT	Mutant			WT	Mutant	WT	Mutant		
V190 A	123.7 ± 5.65	5	9.95 ± 0.06	10.35 ± 0.12^*^	37.66 ± 7.22	37.34 ± 7.90	−0.49 ± 0.15^#^	5	8.48 ± 0.16	9.45 ± 0.06^*^	25.36 ± 5.56	26.34 ± 6.43	−1.08 ± 0.23^#^	4
H194 A	87.8 ± 10.2	5	9.62 ± 0.09	9.31 ± 0.10	30.06 ± 3.55	27.42 ± 3.24	0.41 ± 0.13	4	8.13 ± 0.03	8.07 ± 0.17	35.38 ± 4.63	31.48 ± 4.13	0.17 ± 0.20	4
N226 A	130.3 ± 12.0	6	10.09 ± 0.25	10.52 ± 0.18	33.19 ± 10.55	36.90 ± 11.62	−0.59 ± 0.41	4	8.49 ± 0.09	9.30 ± 0.12^*^	29.39 ± 10.45	31.64 ± 10.06	−0.96 ± 0.29^#^	4
Y227 A	99.2 ± 7.53	5	10.10 ± 0.11	9.23 ± 0.15^*^	36.34 ± 8.51	32.99 ± 9.43	0.91 ± 0.26^#^	5	8.32 ± 0.08	7.45 ± 0.09^*^	33.89 ± 12.01	28.92 ± 8.41	0.94 ± 0.25^#^	4
M230 A	86.0 ± 6.77	5	10.06 ± 0.15	9.31 ± 0.09^*^	26.85 ± 5.26	33.98 ± 4.90	0.71 ± 0.22^#^	6	8.46 ± 0.12	7.77 ± 0.13^*^	26.33 ± 7.27	24.18 ± 7.39	0.79 ± 0.27^#^	4
E233 A	74.0 ± 5.60	5	9.86 ± 0.12	9.62 ± 0.15	29.88 ± 5.30	27.47 ± 2.75	0.41 ± 0.24	4	8.46 ± 0.11	7.92 ± 0.10^*^	20.54 ± 4.71	16.76 ± 3.84	0.75 ± 0.23^#^	6
H295 A	73.1 ± 7.67	6	9.72 ± 0.04	8.27 ± 0.09^*^	35.13 ± 5.18	18.04 ± 3.35^*^	1.88 ± 0.15^#^	4	8.17 ± 0.03	7.86 ± 0.07^*^	35.69 ± 4.44	21.66 ± 2.93^*^	0.66 ± 0.12^#^	4
I298 A	105.3 ± 9.33	5	10.15 ± 0.09	9.51 ± 0.11^*^	34.96 ± 7.39	28.51 ± 7.50	0.71 ± 0.22^#^	5	8.33 ± 0.10	7.12 ± 0.05^*^	31.42 ± 5.56	20.78 ± 4.86	1.36 ± 0.18^#^	5
L302 A	105.4 ± 6.14	5	9.99 ± 0.10	9.26 ± 0.07^*^	39.28 ± 7.64	34.77 ± 8.22	0.76 ± 0.16^#^	4	8.40 ± 0.08	8.02 ± 0.04^*^	40.46 ± 7.54	29.48 ± 3.82	0.49 ± 0.14^#^	4
N305 A	95.5 ± 4.42	6	10.08 ± 0.06	7.60 ± 0.12^*^	31.16 ± 8.65	19.35 ± 5.94	2.70 ± 0.23^#^	5	8.32 ± 0.15	6.52 ± 0.11^*^	33.54 ± 3.28	8.93 ± 1.52^*^	2.39 ± 0.21^#^	3
L345 A	96.1 ± 6.69	6	10.18 ± 0.15	8.29 ± 0.08^*^	27.99 ± 8.60	19.96 ± 7.11	2.05 ± 0.28^#^	5	8.41 ± 0.16	7.46 ± 0.53	21.38 ± 5.24	6.26 ± 1.57^*^	1.50 ± 0.61	4
E348 A	93.1 ± 0.93	3	10.12 ± 0.07	9.82 ± 0.01^*^	21.66 ± 2.65	22.37 ± 5.74	0.32 ± 0.13	3	8.08 ± 0.10	8.09 ± 0.07	18.79 ± 1.60	16.38 ± 2.61	0.08 ± 0.14	3
F349 A	90.1 ± 6.70	5	9.89 ± 0.20	9.98 ± 0.14	28.30 ± 6.91	26.49 ± 6.13	−0.02 ± 0.30	4	8.13 ± 0.06	8.60 ± 0.21	36.89 ± 4.91	32.33 ± 5.55	−0.37 ± 0.25	4
H370 A	83.2 ± 12.5	5	9.61 ± 0.07	8.89 ± 0.08^*^	34.35 ± 5.38	32.71 ± 5.03	0.82 ± 0.16^#^	4	8.19 ± 0.10	8.25 ± 0.06	28.38 ± 6.10	30.30 ± 4.66	−0.01 ± 0.18	4
I371 A	106.7 ± 13.1	5	10.02 ± 0.07	10.27 ± 0.18	31.61 ± 5.98	30.94 ± 5.35	−0.27 ± 0.25	5	8.52 ± 0.13	8.68 ± 0.08	26.16 ± 6.47	24.52 ± 6.24	−0.16 ± 0.24	4
M373 A	97.7 ± 9.19	6	9.89 ± 0.05	9.98 ± 0.08	39.87 ± 7.26	39.27 ± 7.94	−0.07 ± 0.16	4	8.32 ± 0.08	7.84 ± 0.13^*^	40.18 ± 8.09	40.16 ± 8.95	0.49 ± 0.21	4
H374 A	139.7 ± 16.5^*^	6	9.93 ± 0.02	10.20 ± 0.09^*^	35.40 ± 7.26	41.79 ± 11.17	−0.49 ± 0.20	4	8.43 ± 0.14	10.44 ± 0.20^*^	26.98 ± 9.13	37.28 ± 12.87	−2.30 ± 0.37^#^	5
Q376 A	94.3 ± 7.57	6	9.66 ± 0.08	9.47 ± 0.05	30.05 ± 3.55	29.17 ± 4.64	0.22 ± 0.14	4	8.23 ± 0.09	8.07 ± 0.21	28.69 ± 6.08	29.67 ± 5.15	0.17 ± 0.27	4

All data are mean ± S.E.M. The number of independent experiments for each mutant is shown. ^*^p < 0.05 vs WT. For C.S.E. data, statistical analysis was by one-way ANOVA, followed by Dunnett’s test. For pEC_50_ data, statistical analysis was by unpaired *t* test. ^#^95% confidence limits exclude 0.

### 3.3 Effect of CLR alanine substitution on cAMP production

**Fig. 3:**
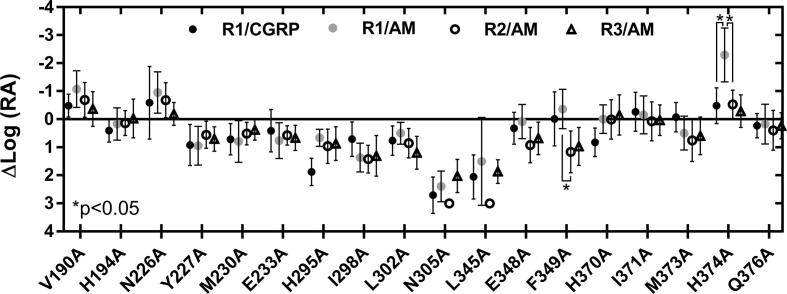
Δlog(RA) values for all ligands at all receptors tested, providing a global summary of the data. 95% CI are shown. Multiple comparisons of the values for each mutant are shown, where statistically significant.

**Fig. 4:**
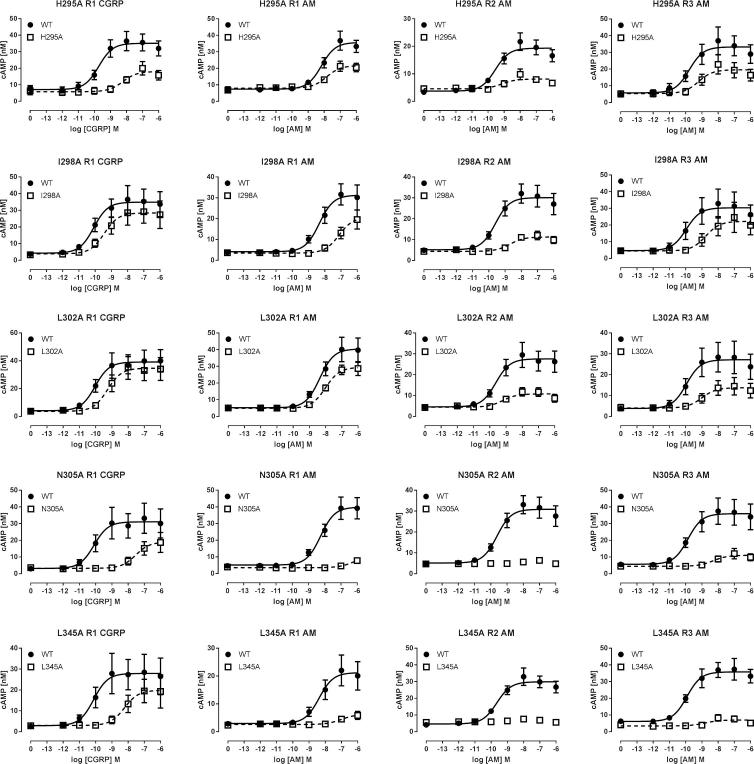
Concentration-response curves for selected mutants with effects that were mostly independent of ligand or RAMP. Each point is the combined mean ± s.e.m. from 4 to 6 independent experiments, each performed with triplicates.

**Fig. 5:**
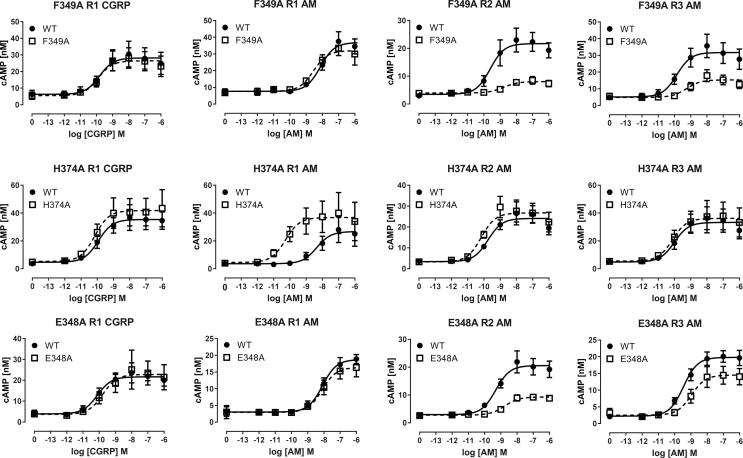
Concentration-response curves for mutants with RAMP or ligand-dependent effects. Each point is the combined mean ± s.e.m. from 3 to 5 independent experiments, each performed with triplicates.

pEC_50_, E_max_ and Δlog(RA) values are shown in Table 1, Table 2, Table 3 for the CLR/RAMP1, 2 and 3 heteromers. Differences from WT in Δlog(RA) values are illustrated in Fig. 3 and concentration response curves for a range of mutants are shown in Fig. 4, Fig. 5. Basal values were not significantly different between the alanine substitutions and WT receptor (data not shown).

**Table 2:** Cell surface expression (C.S.E.) and cAMP production for RAMP2/CLR mutants, using AM as agonist.

Mutant	C.S.E.	n	pEC_50_		E_max_ cAMP (nM)		ΔLog(RA)	n
			WT	Mutant	WT	Mutant		
V190 A	99.1 ± 3.39	5	9.69 ± 0.10	10.30 ± 0.15^*^	25.47 ± 4.06	28.40 ± 6.31	−0.69 ± 0.22^#^	5
H194 A	99.5 ± 4.02	5	9.66 ± 0.12	9.53 ± 0.07	18.52 ± 1.20	18.24 ± 1.52	0.14 ± 0.16	4
N226 A	109.6 ± 6.60	6	9.79 ± 0.09	10.44 ± 0.15^*^	26.19 ± 6.26	25.63 ± 6.07	−0.68 ± 0.24	4
Y227 A	110.9 ± 4.33	5	9.71 ± 0.07	9.22 ± 0.10^*^	26.26 ± 4.68	20.56 ± 3.90	0.55 ± 0.17^#^	6
M230 A	95.5 ± 13.7	5	9.90 ± 0.08	9.32 ± 0.09^*^	16.82 ± 0.49	20.81 ± 2.08	0.50 ± 0.15^#^	4
E233 A	78.0 ± 9.01	5	9.79 ± 0.03	9.60 ± 0.06^*^	22.56 ± 2.17	11.96 ± 2.31^*^	0.57 ± 0.13^#^	4
H295 A	74.7 ± 7.37	6	9.55 ± 0.01	9.09 ± 0.21	19.33 ± 2.64	8.19 ± 0.69^*^	0.96 ± 0.24^#^	4
I298 A	88.3 ± 11.6	5	9.61 ± 0.05	8.67 ± 0.12^*^	30.15 ± 4.84	11.36 ± 1.63^*^	1.42 ± 0.18^#^	4
L302 A	90.6 ± 9.48	5	9.66 ± 0.05	9.26 ± 0.12^*^	27.60 ± 5.26	10.81 ± 1.97^*^	0.85 ± 0.19^#^	4
N305 A	72.4 ± 11.2	6	9.64 ± 0.08	N.C.	28.40 ± 5.54	N.C.	–	4
L345 A	89.9 ± 5.34	6	9.67 ± 0.06	N.C.	29.92 ± 3.88	N.C.	–	4
E348 A	101.1 ± 7.33	3	9.33 ± 0.08	8.75 ± 0.06^*^	20.59 ± 3.29	9.32 ± 0.93^*^	0.92 ± 0.15^#^	3
F349 A	95.9 ± 3.57	5	9.49 ± 0.21	8.78 ± 0.13^*^	21.96 ± 2.89	8.06 ± 1.06^*^	1.16 ± 0.27	3
H370 A	82.9 ± 14.4	5	9.53 ± 0.15	9.59 ± 0.15	21.35 ± 2.14	22.15 ± 2.30	0.01 ± 0.25	4
I371 A	96.2 ± 18.7	5	9.75 ± 0.08	9.72 ± 0.15	22.55 ± 4.07	21.43 ± 4.88	0.07 ± 0.25	4
M373 A	105.7 ± 15.6	5	9.72 ± 0.12	9.10 ± 0.15^*^	27.06 ± 6.50	19.04 ± 3.79	0.75 ± 0.27	4
H374 A	109.4 ± 7.32	6	9.78 ± 0.10	10.23 ± 0.11^*^	24.25 ± 4.07	26.84 ± 5.00	−0.53 ± 0.20	5
Q376 A	96.6 ± 11.5	6	9.66 ± 0.20	9.41 ± 0.13	20.67 ± 2.44	15.46 ± 1.44	0.39 ± 0.27	4

All data are mean ± S.E.M. The number of independent experiments for each mutant is shown. N.C. No curve; the response was too weak to fit curves to the data. ^*^p < 0.05 vs WT. For C.S.E. data, statistical analysis was by one-way ANOVA, followed by Dunnett’s test. For pEC_50_ data, statistical analysis was by unpaired *t* test. ^#^95% confidence limits exclude 0.

**Table 3:** Cell surface expression (C.S.E.) and cAMP production for RAMP3/CLR mutants, using AM as agonist.

Mutant	C.S.E.	n	pEC_50_		E_max_ cAMP (nM)		ΔLog(RA)	n
			WT	Mutant	WT	Mutant		
V190A	105.2 ± 8.46	5	9.99 ± 0.05	10.29 ± 0.12	35.29 ± 10.05	39.38 ± 9.36	−0.36 ± 0.22	4
H194A	99.6 ± 9.36	5	9.95 ± 0.18	10.01 ± 0.08	36.13 ± 7.87	33.79 ± 5.16	−0.03 ± 0.25	4
N226A	99.4 ± 3.76	5	10.07 ± 0.02	10.26 ± 0.06^*^	33.55 ± 7.34	34.01 ± 6.46	−0.19 ± 0.15	5
Y227A	94.5 ± 7.90	5	9.97 ± 0.06	9.35 ± 0.07^*^	35.68 ± 6.43	31.25 ± 6.01	0.70 ± 0.16^#^	4
M230A	101.4 ± 5.91	5	9.91 ± 0.07	9.63 ± 0.05^*^	31.09 ± 5.25	24.70 ± 3.27	0.37 ± 0.13	5
E233A	91.3 ± 15.4	5	10.02 ± 0.05	9.60 ± 0.08^*^	21.45 ± 4.21	13.54 ± 1.83	0.66 ± 0.16^#^	6
H295A	85.8 ± 9.0	5	9.77 ± 0.13	9.20 ± 0.07^*^	33.45 ± 6.56	19.60 ± 4.59	0.87 ± 0.22^#^	5
I298A	100.3 ± 14.1	5	9.90 ± 0.12	8.73 ± 0.09^*^	30.42 ± 7.73	22.25 ± 7.14	1.30 ± 0.26^#^	4
L302A	97.6 ± 5.64	5	9.95 ± 0.05	9.06 ± 0.10^*^	27.16 ± 7.05	13.80 ± 3.84	1.19 ± 0.21^#^	5
N305A	103.1 ± 20.7	5	9.86 ± 0.09	8.34 ± 0.07^*^	36.04 ± 7.60	11.06 ± 2.56^*^	2.02 ± 0.21^#^	4
L345A	90.4 ± 7.12	5	9.91 ± 0.08	8.80 ± 0.03^*^	35.89 ± 5.16	7.00 ± 1.55^*^	1.86 ± 0.15^#^	4
E348A	92.6 ± 7.99	3	9.46 ± 0.06	8.95 ± 0.06^*^	19.82 ± 2.00	14.55 ± 2.71	0.68 ± 0.13^#^	3
F349A	102.2 ± 13.6	5	9.89 ± 0.14	9.23 ± 0.11^*^	31.83 ± 6.31	15.45 ± 2.95^*^	0.96 ± 0.24^#^	5
H370A	101.2 ± 11.0	5	9.82 ± 0.10	10.00 ± 0.18	38.02 ± 6.22	35.40 ± 7.02	−0.15 ± 0.26	4
I371A	97.0 ± 6.85	5	10.00 ± 0.13	9.96 ± 0.09	17.63 ± 2.88	18.56 ± 2.66	0.03 ± 0.20	5
M373A	114.2 ± 10.9	5	10.03 ± 0.07	9.38 ± 0.14^*^	32.20 ± 8.47	32.40 ± 7.65	−0.65 ± 0.22	4
H374A	113.3 ± 5.78	5	10.00 ± 0.07	10.20 ± 0.10	33.32 ± 7.47	36.22 ± 10.24	−0.29 ± 0.20	4
Q376A	110.7 ± 9.06	5	9.91 ± 0.09	9.66 ± 0.07	35.57 ± 6.57	33.50 ± 5.25	0.23 ± 0.17	4

All data are mean ± S.E.M. The number of independent experiments for each mutant is shown. ^*^p < 0.05 vs WT. For C.S.E. data, statistical analysis was by one-way ANOVA, followed by Dunnett’s test. For pEC_50_ data, statistical analysis was by unpaired *t* test. ^#^95% confidence limits exclude 0.

H194A, I371A and Q376A were the only substitutions not to have had any effect on receptor potency or efficacy. The majority of mutations showed small (<10-fold) reductions in pEC_50_, and Δlog(RA) values; E_max_ values were typically reduced by up to 30%. Generally there were similar trends across all receptors and ligands. The largest effects on cAMP were seen at I298A, L302A, N305A, and L345, although in all of these the most prominent effects on E_max_ occurred with RAMPs 2 and 3 (Fig. 4). H295A showed reductions in E_max_ of around 50% for all ligand/RAMP combinations, although the reduction in pEC_50_ was particularly marked for CGRP (Fig. 4). E348A and F349 demonstrated clear RAMP-dependent effects; signaling at the RAMP2 and RAMP3 complexes was curtailed but was barely changed for RAMP1 (Fig. 5). For H374A, the mutation was without major effect on any receptor/ligand combination apart from AM at the CLR/RAMP1 complex, where potency was increased by 100-fold (Fig. 5). For many mutants the effects were observable in changes in relative activity, although this is not generally a sensitive indicator of changes due to the accumulation of errors involved in the calculation of this parameter. V190A and N226A also caused small increases in ligand potency for AM at the CLR/RAMP1 and RAMP2 receptors. For N226A there was also a small effect at CLR/RAMP3.

### 3.4 Radioligand binding

**Fig. 6:**
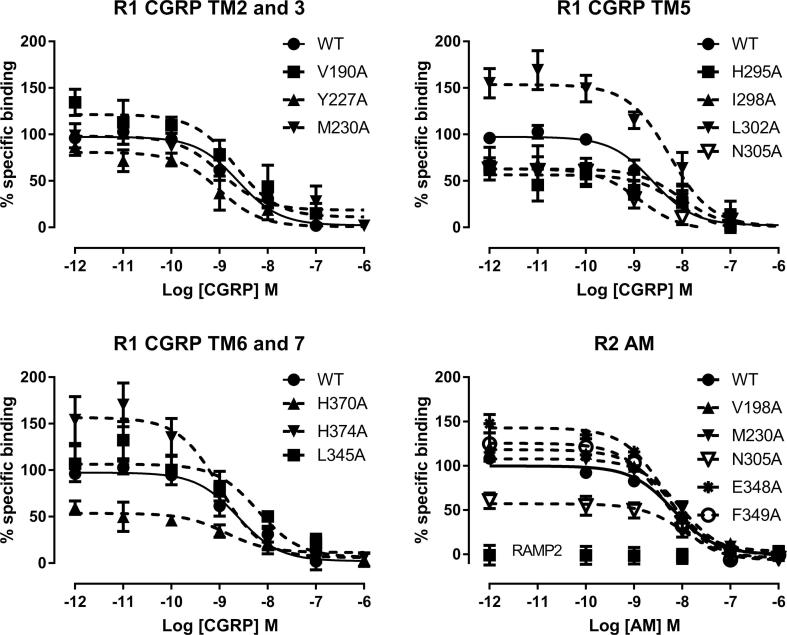
Radioligand binding of mutants. Displacement of ^125^I-CGRP by CGRP at CLR/RAMP1 or displacement of ^125^I-AM by CGRP at CLR/RAMP2. For AM, binding with RAMP2 alone is also shown. Values are mean ± s.e.m. of 4–6 independent determinations for CGRP or 3 independent determinations for AM.

The residues that showed changes in potency for CGRP at the CLR/RAMP1 complex were examined by competition radioligand binding, using ^125^I-CGRP and competition with unlabelled CGRP (Fig. 6). There were reductions in the binding relative to WT for H295A, I298A, L302A, N305A and H370A (Fig. 6, Table 4). A more limited characterization was carried out using ^125^I-AM (Fig. 6, Table 4). Despite substantial reductions in cAMP production, E348A and F349A retained the ability to bind AM at the AM_1_ receptor. Furthermore, the use of ^125^I-AM confirmed that H374A had increased affinity for AM when expressed with RAMP1 (specific binding of ^125^I-AM, 78 ± 208 dpm/mg to WT CLR/RAMP1; 5243 ± 112 dpm/mg to H374A CLR/RAMP1). There was also an increase in CGRP binding at H374A CLR/RAMP1.

**Table 4:** Radioligand binding to RAMP1/CLR and RAMP2/CLR receptors.

	pIC_50_	% Ligand bound	n
**RAMP1/CLR**			
WT	8.92 ± 0.11	100	6
V190A	8.60 ± 0.35	149.2 ± 24.2	5
Y227A	8.52 ± 0.36	84.0 ± 9.6	5
M230A	9.25 ± 0.20	74.6 ± 12.7	6
H295A	8.62 ± 0.30	53.3 ± 13.6^*^	4
I298A	8.31 ± 0.24	62.5 ± 9.1^*^	5
L302A	8.25 ± 0.19	171.4 ± 20.5^*^	6
N305A	8.82 ± 0.18	66.8 ± 9.1^*^	5
L345A	8.43 ± 0.12	123.9 ± 22.8	5
H370A	8.59 ± 0.37	53.8 ± 7.7^*^	5
H374A	8.59 ± 0.27	213.4 ± 35.4^*^	6

**RAMP2/CLR**			
WT	8.12 ± 0.08	100	3
V198A	8.24 ± 0.18	116.2 ± 3.7^*^	3
M230A	8.04 ± 0.06	109.4 ± 3.7	3
N305A	7.96 ± 0.09	62.7 ± 4.7^*^	3
E348A	8.38 ± 0.13	147.9 ± 2.9^*^	3
F349A	8.37 ± 0.07	132.3 ± 4.3^*^	3

The% ligand bound is the amount of ^125^I-CGRP (RAMP1/CLR) or ^125^I-AM (RAMP2/CLR) bound (taken as the span of the displacement curves from Prism) and normalized to the values for the WT receptor. *, 95% CI does not overlap 100.

### 3.5 V190A, N226A and H374A alter helix packing in simulations

**Fig. 7:**
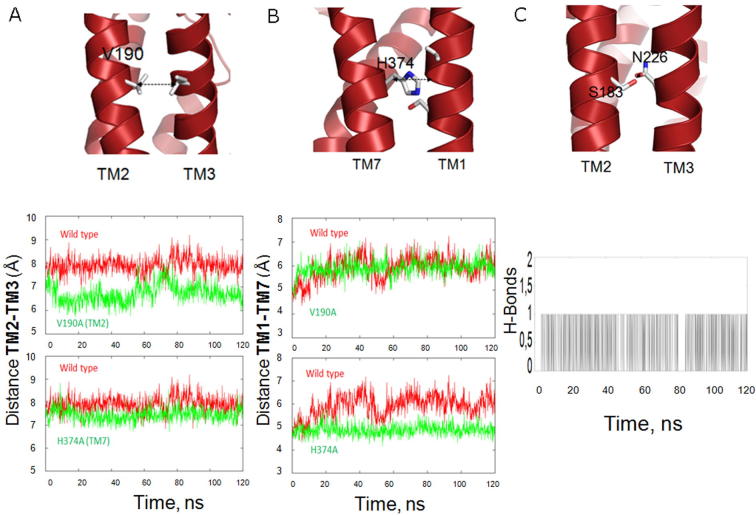
Interactions between transmembrane (TM) helices as seen in a molecular dynamics simulation of the TM domain of inactive CLR (red). Changes in inter-helical distances for the V190A (A) and H374A (B) mutants is associated with an increase in potency for some ligand/RAMP combinations. The TM2 – TM3 distance is shown for wild-type and V190A and for wild-type and H374A. The TM1 – TM7 distance is shown for wild-type and V190A and for wild-type and H374A. Also shown is hydrogen bond formation between S183 and N225 (C). (For interpretation of the references to color in this figure legend, the reader is referred to the web version of this article.)

V190A, N226A and H374A all increase agonist potency, particularly for RAMP1 with AM. To elucidate possible mechanisms for the effect of these mutations on the packing of the TM helices, molecular dynamics simulations were carried out on models of WT, and single point mutants V190A and H374A of CLR, all in the inactive form. The distance between TM2 and TM3 (Cα of residue V190 – Cα of residue I218) and between TM7 and TM1 (Cα of residue G148 – Cα of residue H374), was monitored. Fig. 7 shows that mutant V190A (but not H374A) decreases the distance between TM2 and TM3 while mutant H374A (but not V190A) decreases the distance between TM7 and TM1. In addition, residue N226 located in TM3 forms a stable hydrogen bond with S183 in TM2, thus its function may be to calibrate the distance between TM2 and TM3, analogous to V190. The models of these mutants predict closer packing of the TM helices and this may underlie the increased potency of AM, by allowing better contacts between residues in the binding pocket and the peptide.

### 3.6 The active/inactive transition in CLR and the predicted G protein binding pocket

**Fig. 8:**
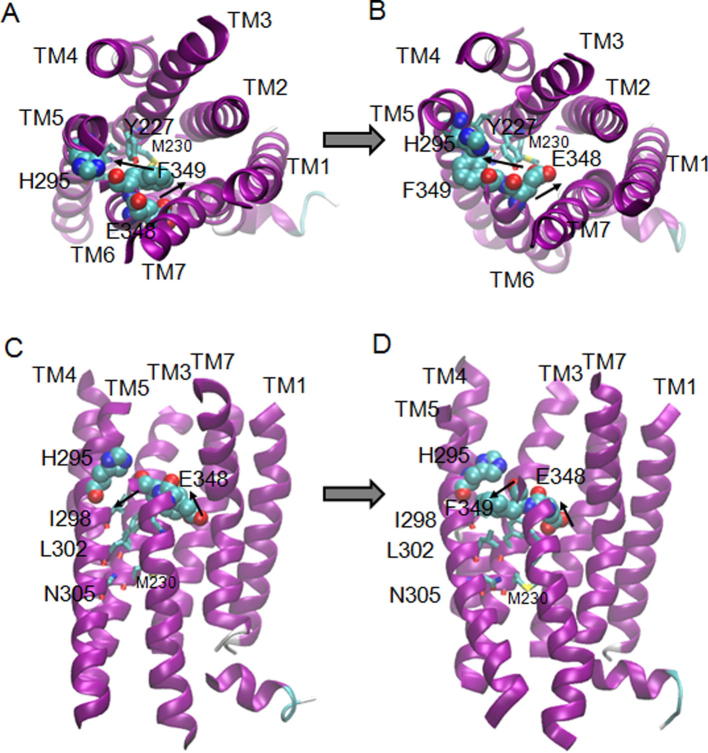
Rotation of residues in the transition from the inactive (A, C) to the active (B, D) transmembrane bundle of CLR (purple). Views looking towards the extracellular face of the receptor are shown in A and B; views from the side of the receptor are shown in C and D. (For interpretation of the references to color in this figure legend, the reader is referred to the web version of this article.)

To reveal the sequence of events necessary within the TM bundle to create the binding pocket for Gs, a molecular dynamics simulation was used to study the transition in the TM bundle between the active and inactive forms of CLR. The simulation was set up without RAMPs or bound ligand. During the course of the simulation, TM helices 5 and 6 pivoted around TM3 to expose the cytoplasmic G protein binding pocket. Y227, M230, I298, L302, N305, L345 and F349 collectively formed the pivot for the helix rearrangement and there were concerted movements of all of these residues, with rotations of H295 and F349 (Supplementary animation; Fig. 8). These were accompanied by movement of E348 from facing Q376A to pointing to the middle of the TM bundle. At the extracellular face of the receptor, there were movements of TM7 to approach TM1 and TM5 towards TM4.

**Fig. 9:**
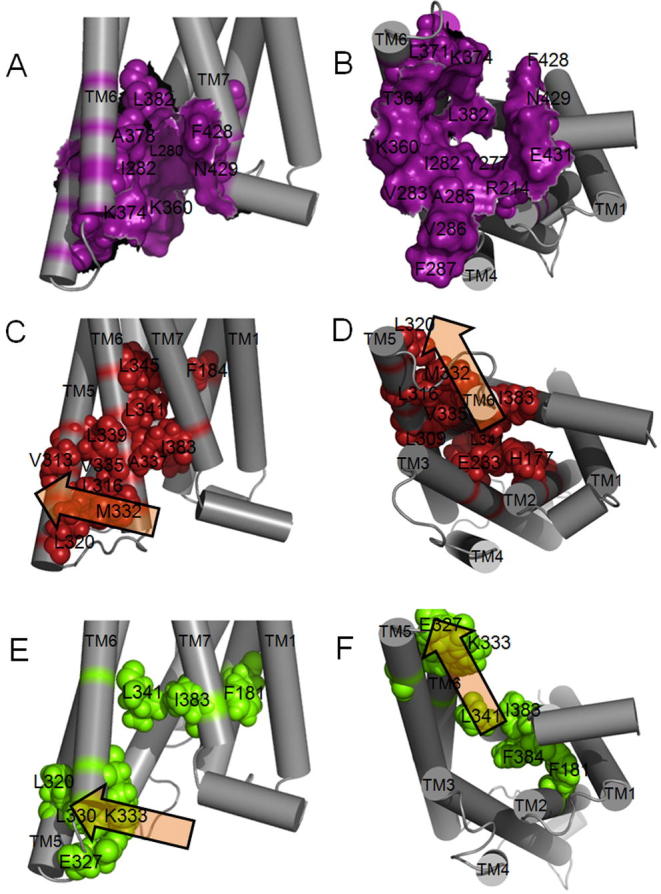
CLR residues that form the predicted G protein binding pocket and those which undergo a rearrangement upon activation. The surface of residues that form the G protein binding site in the active state model with RAMP 1 is colored purple, viewed from the receptor side in A and from the intracellular side in B. Residues that contact each other in the inactive state TMD model of CLR are shown in spacefill colored red (C,D), while those that contact each other in the active state model with RAMP 1 are in spacefill and colored green (E,F). The residues at the G protein interface are **R173** (ICL1), **H177** (TM2), Y236, L237, L240, (TM3), I241, V242, A244, **V245**, **F246**, (ICL2), **I312**, V315, **L316** (TM5), *K319*, T323, L330 (ICL3), A332, *K333*, A337, **L341** (TM6), F387, **N388** (TM7), G389, **E390** (H8). Residues in bold disrupt Gs coupling when mutated to alanine, residues in italics have no effect. (For interpretation of the references to color in this figure legend, the reader is referred to the web version of this article.)

In Fig. 9, the residues that are predicted to be involved in forming the G protein binding pocket in the fully active CGRP receptor (CLR/RAMP1/CGRP/Gs) complex are illustrated. Sixteen of these have been mutated to alanine and in nine cases this disrupts coupling to Gs [13], [14], [50]. As the majority of the contacts are hydrophobic, we would not expect alanine substitution to disrupt all interactions. Table 5 shows the interactions that are made (present in active conformation) and broken (present in inactive conformation); there is also mutagenic support for eight of these out fourteen tested.

## 4 Discussion

Gs is the most prominent coupling pathway for class B GPCRs, including CLR, and is amenable to modeling, given the availability of a crystal structure of a GPCR with this G protein (pdb code: 3SN6) [39]. Here we address the Gs-linked activation mechanism within the TM bundle of CLR, the extent to which RAMPs influence this mechanism and whether two endogenous ligands, CGRP and AM engage this in different ways. The study has identified an upper ring of residues in the TM bundle which are particularly influenced by RAMPs as well as networks of hydrophilic and hydrophobic amino acids.

### 4.1 An upper group of residues transmit changes from the agonist binding pocket to the TM bundle

**Fig. 10:**
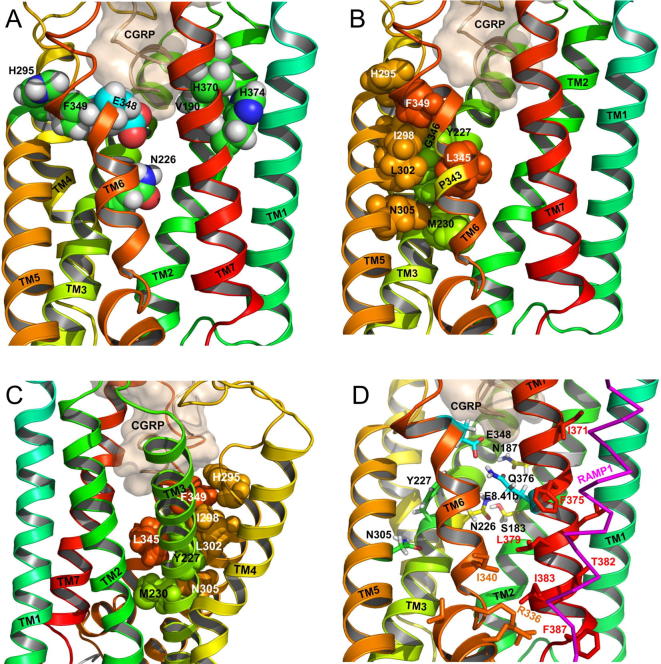
Key residues within the TM bundle of the active CLR in complex with CGRP and RAMP1. CGRP is wheat colored, the RAMP TM helix is magenta. CLR is colored in rainbow mode from cyan (TM1) to red (TM7). Residue labels are colored for clarity only. A) The position of the ring residues within the CLR TM bundle. B and C). The position of selected CLR hydrophobic residues on TM3, TM5 and TM6 shown from opposite sides of the receptor. In B, the residues are shown from the same side of the receptor as in (A) and (D); in B and C the residues are colored by helix. The positions of P343 and G346 are marked in yellow on TM6 in B. Y227 and L345, formally equivalent to the class A connector region (I^3.40^ and F^6.44^ in the β_2_-AR), are in close proximity. D). The position of selected CLR hydrophilic residues on TM2, TM3, TM5, TM6 and TM7. CGRP is wheat colored; the RAMP TM helix is magenta. Residues are shown in stick form to allow the positions of potential coulombic interactions to be visualized; mutually interacting residues are shown in the same color. Residues that interact with RAMP1 in this average structure (structure with the lowest RMSD to the average structure, as determined by the visual molecular dynamics software) are shown in line form and colored according to the helix color; residues that form persistent interactions (i.e. <5 Å in more than 80% of the frames in all 4 MD simulations) are identified by their residue number. (For interpretation of the references to color in this figure legend, the reader is referred to the web version of this article.)

V190, N226, H295, H374, E348 and F349 are at a similar level in the TM bundle and face each other (Fig. 10). Just above these lies H370. Mutation of all of these alters receptor activation, but in a RAMP and ligand-dependent manner. Collectively they form a broad ring, linking the ECLs with the TM bundle of CLR and combine with the residues of the ECLs in influencing the binding of AM and CGRP in a RAMP-dependent manner [51], [8]. Our data suggest that CGRP (Fig. 8, Fig. 10) and AM (not shown) make contacts to both the ECLs and the upper part of the TM bundle, similar to those postulated in models and structures of calcitonin, CRF, glucagon and GLP-1 binding to their receptors [61], [59], [46], [32], [63], [44]. Some residues within this upper cluster such as H370 may contact the peptide directly (Fig. 10). RAMPs act on these residues to confer pharmacological specificity, either by direct contacts or by allosteric actions [51].

The precise mechanism of action of these residues is speculative, although in broad terms it is easy to see how movement of the ECLs in response to agonist binding [51] could be transmitted to the TM bundle via residues such as H295 (TM5), E348 (TM6) and F349 (TM6), and H374 (TM7). In our model, a rotameric shift of the imidazole sidechain of H374 would require movement of the backbone of ECL3 in the vicinity of H370, thus potentially changing ligand binding as seen in our data (Fig. 10). It is remarkable that a single amino acid change at H374 can have such a dramatic effect on the pharmacology of the receptor, driving a potent AM response in a CGRP receptor. We have previously argued that the orientation of ECL3 may be an important determinant of ligand selectivity and that this is modulated by RAMPs [51]. The ability of mutation of V190, N226 and H374 to increase AM potency suggests that the upper region of the TM2/3/7 interface is particularly important in peptide binding and selectivity, perhaps by controlling the conformation of ECL3 via closer contacts within the TM bundle. Interestingly, TM2/7 interactions in this region have been predicted to act as a switch in the CRF1R [42].

### 4.2 Residues in TMs 3, 5 and 6 form a hydrophobic cluster allowing helix tilting

The mutagenesis data identified a cluster of residues in TMs, 3, 5 and 6, which act in a RAMP-independent fashion to facilitate CLR activation. The modeling suggests that collectively they form a pivot region, to allow the movement of TM5 and 6 relative to TM3 during the transition from an inactive to an active state, similar to mechanisms described in class A GPCRs, discussed further below. There are extensive rearrangements between Y227, M230, I298, L302, N305 and L345 during the course of this process, which effectively amounts to the resetting of a hydrophobic latch. Y227 (Y/F), M230 (M/L/I), I298 (M/I/V/L), L302 (L/I/V), N305 and L345 (L/F) (Fig. 10) are conserved throughout class B GPCRs suggesting that the TM3/5/6 latch/pivot might be a general mechanism. L345 lies between the absolutely conserved G346 and the highly conserved P343 [12], which allow independent movement of the intracellular and extracellular ends of TM6 (Fig. 10). The rearrangements also involve H295 and F349, linking changes in the latch to the upper ring of amino acids and so potentially to ligand binding.

This network has similarities to the “connector region” in class A GPCRs [18], [36], [31]. These are a collection of hydrophobic residues in TMs 3, 5 and 6. The key players in the class A connector region are I^3.40^ and F^6.44^ [18], with other key residues namely P^5.50^, W^6.48^ and I^6.40^ also part of a central hydrophobic core. Analysis of crystal structures and simulations shows distinct changes in the β_2_-adrenergic receptor, e.g. the rotational movement of TM6 on activation means that M272^6.41^ and F282^6.44^ face TM5 rather than TM3 as in the inactive state, though the configuration of these residues is less changed on activation in the muscarinic acetylcholine M_2_ receptor. A number of these key hydrophobic residues are group conserved in both class A and class B GPCRs [50], but speculation on how this mechanism transfers to class B is complicated by the observation that the equivalent residues form a comparable well-defined cluster in the glucagon receptor X-ray structure [43], [26] but not in the CRF1R X-ray structure [23]. However, mutagenesis has identified a hydrophobic interaction between 3.40, 6.44 and 6.48 in the CRF1R [46]. Analysis of the molecular dynamics simulations showed that the switch from TM3 interactions to TM5 interactions on activation occurs lower down TM6 in CLR (at T338 rather than L341). The origin of this difference is probably because P343 in CLR is two turns lower in class B GPCRs than in the well conserved P5.50 in class A [12]. Moreover, TM6 is much straighter in the class B inactive structures [43], [23], [26] than in comparable class A structures. This straight conformation may be influenced by the binding of antagonists deep within the TM bundle [23] or at the intracellular end of TM6 [26], and as discussed below, this ensures that the activation process involves much more than rigid body rotation of TM6.

While we believe it is possible to identify considerable conservation of mechanisms involving the hydrophobic networks both between and within GPCR families, the data also show how elements of this network are uniquely adapted in individual receptors. Thus residue 6.48 is a key part of the hydrophobic network in class A GPCRs and the CRFR1 [46]; in the GIPR and GLP-1 receptors it takes part in a hydrophilic network [15], [58], [59], [60], but it only appears significant on alanine mutation for CLR/RAMP2 and RAMP3, not RAMP1. Furthermore, E354A^6.48^ increases GIP potency at the GIPR showing the main effect of the residue is to stabilize the inactive state of the receptor but for CLR/RAMP2 and RAMP3, mutation of F349^6.48/6.53b^ impairs activation [15], [46].

### 4.3 Hydrophilic networks

**Fig. 11:**
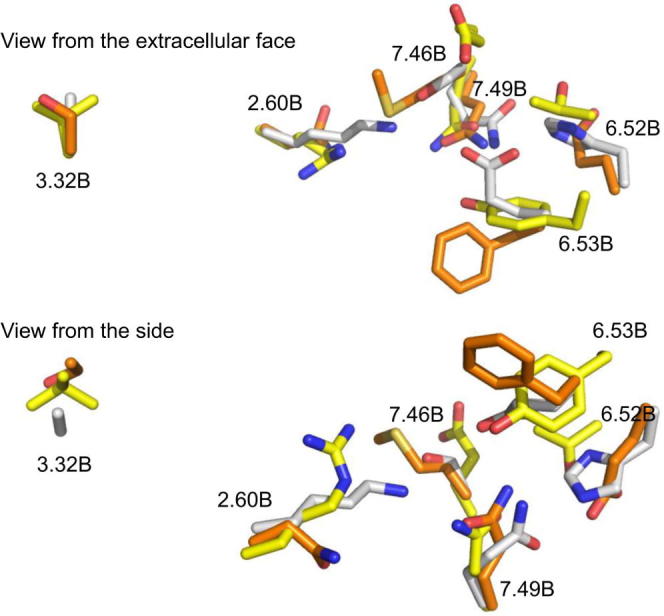
Comparison of the upper TM hydrophilic network in the CRF1 receptor (4K5Y, yellow), the glucagon receptor expressed as a fusion protein (4L6R, white) and the inactive structure of CLR (orange). (For interpretation of the references to color in this figure legend, the reader is referred to the web version of this article.)

Previous work on the mechanism of activation of class B GPCRs has considered hydrophilic interactions, mediating inter-helical contacts, particularly a network in the central region of the TM bundle between residues 2.60^b^, 3.43^b^, 5.50^b^, 6.52^b^, 7.49^b^ and 7.57^b^ [60], [58], [15] (Fig. 11). In the current study, N226, N305A, E348A and Q376A have been examined (Fig.10D). In class B GPCRs, residue N305 is highly conserved as an asparagine; the modeling of CLR suggests it hydrogen bonds to the backbone carbonyls of Y227 and M230; similar interactions have been proposed in the CRFR1 [46]. Alanine substitution of N^5.50b^ caused a reduction in agonist potency in the CRF1 [42], GIP [15] and GLP-1 [60] receptors. However the reduction observed in these receptors was slight compared with the large effects in the CGRP and AM receptor. Thus the role of N305 in CLR may be particularly adapted to that receptor. In the β_2_-adrenergic receptor the corresponding residue (M215) is proposed to stabilize the connector region [31].

The modeling suggests that N226, S183 and N187 are part of a hydrogen bonded network in both the inactive and active forms of CLR (Fig.10D). However, on activation, this is potentially expanded to include E348 and Q376, which previously formed a separate pair (Fig. 8). There is linkage between the movement of E348 and the rearrangement of TMs 3, 5 and 6. In CLR, in the inactive receptor, E348 is held away from the hydrophilic network by F349. As F349 swings towards TMs 3 and 5, E348 can move to a more central region, where it can engage with the hydrophilic network (Fig. 8 and Fig.10D). The retention of AM binding, coupled with loss of function, supports the hypothesis that E348 and F349 have a greater role in the CLR activation mechanism than directly in ligand binding, an idea supported by the CTR cryo-EM structure [32], but clearly this is also modulated by RAMPs, perhaps ultimately via interactions with ECL3 and TM7 [51], [54]. No effect was observed with alanine mutagenesis of Q376, or, for CGRP and RAMP1, N226 [50]. This may simply mean that in CLR, the Gs-coupled active form of the receptor is stabilized by multiple contacts so the roles of these two residues are not crucial.

As with the hydrophobic network of contacts, there are features of CLR that are distinct from other GPCRs such as the GIPR and GLP-1R. As previously noted, 6.48/6.53b is hydrophobic in CLR and also CTR; by contrast the adjoining 6.47/6.52b is hydrophilic. Position 7.46b is also hydrophobic in CLR and CTR (M/I) and so also exerts its effects sterically; consequently at the upper surface of these receptors there are modification of inter-residue networks (see also [15]) and it is interesting that these networks are also subject to modulation by RAMPs. There are other examples of the replacement of conserved hydrophilic residues for hydrophobic ones in CLR; 7.57b is F in CLR but Y in every other receptor and in the EVxxE motif of H8, the final E is I.

### 4.5 G protein binding

Of the eleven interhelical contacts that are changed during formation of the predicted G protein binding pocket (Table 5), the largest single group are between TM5 and 6, reinforcing the role for this interface as a key driver in the opening of the Gs binding pocket. The second largest group is between bases of TMs 6 and 7, indicating how the change might be propagated.

**Table 5:** Main residue rearrangements upon activation and opening of the G protein binding pocket of CLR.

Residue 1	Residue 2	Conformation
**H177**^2.50b^	**E233**^3.50b^	Inactive
**L302**^5.47b^	V350^6.54b^	Inactive
L309^5.54b^	L339^6.47b^	Inactive
**N311**^5.56b^	R314^5.59b^	Inactive
V313^5.58b^	*L339*^6.43b^	Inactive
**L316**^5.61b^	V335^6.39b^	Inactive
*K319*^ICL3^	Y331^ICL3^	Inactive
*L320*^ICL3^	M332^ICL3^	Inactive
A337^6.41b^	I383^7.56b^	Inactive
**L345**^6.49b^	*Y227*^3.44b^	Inactive
**L345**^6.49b^	*F184*^2.57b^	Inactive
**L345**^6.49b^	**L341**^6.45b^	Inactive
**L341**^6.45b^	I383^7.56b^	Active
**F384**^7.57b^	F181^2.54b^	Active
*K333*^6.37b^	E327^ICL3^	Active
*L320*^ICL3^	L330^ICL3^	Active

Residues shown in bold are those where there is mutagenic support for an effect on Gs coupling; those in italics are where mutation has no effect [14], [13], [50]. The contacts were identified from the inactive state TMD and the active state CLR models.

### 4.6 Comparison with the cryo-EM structure of the calcitonin receptor

**Fig. 12:**
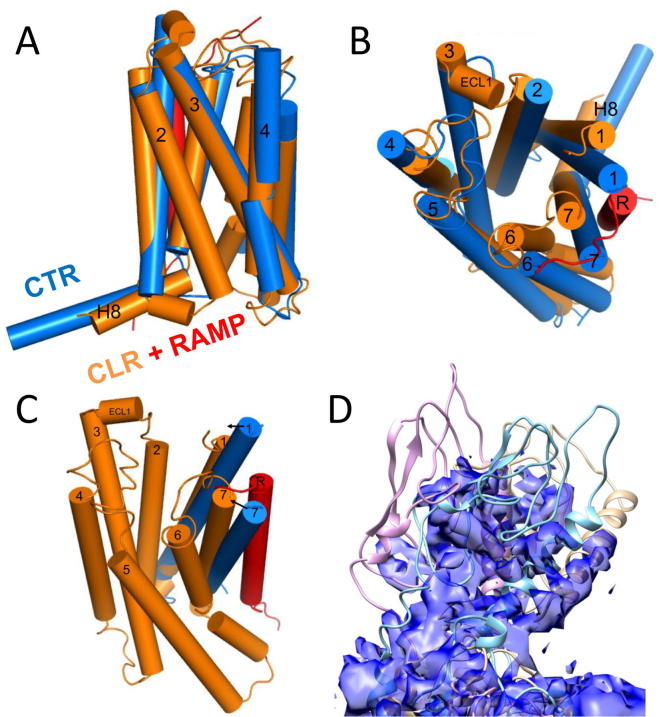
Comparison of the TM domain of CLR (orange) with RAMP1 (red) with the cryo-EM structure of CTR (blue). (A), side view. (B), View from the extracellular side. The presence of the RAMP reorganizes the region around TM1, TM7 and TM6. (C). CLR model (orange) with RAMP1 (red) with superposed TM1 and TM7 of the CTR-EM (blue). The arrows indicate the necessary vector of movement of TM1 and TM7 of CTR to accommodate RAMP1 and prevent a clash. In consequence, TM7 of CLR is located closer to the TM bundle than in the CTR-EM structure. (D). The orientation of the CLR ECD with respect to the electron density of the ECD for the CTR. The surface of the electron density, contoured at 0.014 for the ECD and the top of the TM domain is shown in blue transparent. The starting structure of the CLR/RAMP1 complex, superimposed [37] on the TM domain of the CTR (PDB code 5UZ7) is shown in wheat color (RMSD 2.6 Å over 190 TM domain residues); the final model from the second 500 ns CLR/RAMP1 simulation is shown in cyan (RMSD 3.1 Å over 203 TM domain residues). The final structure from the second 500 ns CLR/RAMP2/AM simulation is shown in purple (RMSD 3.6 Å over 210 TM domain residues). The superposition of the TM domains permits comparison of the ECD regions with respect to the CTR electron density. (For interpretation of the references to color in this figure legend, the reader is referred to the web version of this article.)

Human CLR and the calcitonin receptor (CTR) have identical length loops over the bulk of the structure and share 54% identical residues and 73% similar residues. It is therefore appropriate to compare our model to that of the recent CTR cryo-EM structure [32], even though the CTR structure was generated in the absence of a RAMP. The RMSD of the CLR domain of the CLR/RAMP1 model compared to the CTR cryo-EM structure is about 2.5–3.3 Å over the TM helices, over the course of the 500 ns simulations, as determined using the ccp4mg software [37]. The superposition of a representative CLR TM structure to the CTR TM domain is shown in Fig. 12. The presence of the RAMP causes some degree of re-organization around TM1, TM7 and TM6 and so the RMSD is 1.7–2.1 Å over TM2 – TM5; similar effects were observed in the glucagon receptor – RAMP2 complex [54]. While some of this disparity may also have contributions arising because of the known limitations of homology modeling, some may also be due to the lower resolution of the upper TM region of CTR [32]. Nevertheless, the RMSD is well within the range expected for similar GPCRs [23]. The majority of the structural features in the CTR structure discussed in [32] are similar to those in the CLR/RAMP1 models. For example, CGRP and calcitonin bind to a similar depth just above the conserved central network (N2.60b, N3.43b, Y/F2.57b, Q7.49b, Q6.52b) and the peptides make extensive contacts with the three ECLs, and the peptide helix commences at a similar position (T6 in salmon calcitonin, V8 in CGRP) and the hydrophobic surface of the peptide [51] is facing in a similar direction. While Q14 faces ECL2 in CTR, the corresponding G14 in CGRP is too small to interact with ECL2. T4, like L4 in CTR, points downwards. However, the influence of the RAMP restricting the outward movement of the top of TM6 and hence ECL3 seen in CLR, has perturbed this and other interactions; a consequence is that T4 points more towards TM7/TM2 than TM6. The larger size of the calcitonin disulfide-bonded ring may also have contributed to this difference.

As in CTR, TM6 undergoes significant rearrangement on activation in order to accommodate the C-terminal tail of the G protein. Thus there is a bend of 43° ± 9° (Max 75°) plus a loss of helicity during the MD simulations, which is comparable to the bend of 60° in CLR (which is not restricted by RAMP1). The E^3.50b^ – H^2.50b^ and R^2.46b^ – E^8.41b^ interactions are retained; F^7.57b^ interacts with TM6, despite the loss of an OH group in CLR compared to CTR. Most significantly for this article, the hydrophobic pivot residues (discussed above) are in similar positions.

The orientation of the ECD was particularly difficult to model as the previous cryo-EM data were very low resolution [62]. Nevertheless, TM domains of the initial and two CLR/RAMP MD structures from the 500 ns simulations were structurally aligned to the CTR TM domain (RMSD 2.6 Å, 3.1 Å and 3.6 Å respectively), enabling the fit of the CLR ECD to the CTR cryo-EM electron density to be shown in Fig. 12D. The starting structure fits more closely into the electron density than the average structure as the ECD, like the TM domain, has been perturbed by the RAMP during the MD simulations. Moreover, the variations in the multiple CTR ECD conformations implicit in the electron density maps (Extended data Fig. 5 in reference [32]) are probably comparable with the variations seen for CLR, indicating that CLR and CTR share a degree of flexibility in their ECDs, despite the extreme N-terminus of the ECD interacting with ECL3 in both the CTR structure and the CLR/RAMP1 simulations. Moreover, Jazayeri et al. have shown that the orientation of the ECD to the TM domain may be ligand dependent [27]. A corollary of this is that errors in modeling the ECD could affect interactions within the TM domain. Nevertheless, these variations (Fig. 12D) are relatively small compared to those seen in the glucagon receptor ECD in the presence of an antibody [63]. Given the caveat that the electron density in the ECD is too low a resolution to permit the ECD to be reliably fitted to this density, the maps nevertheless shows that the CLR ECD adopts a reasonable orientation.

### 4.7 Conclusion

Molecular models of CLR have been generated to permit interpretation of mutagenesis data of key residues within the TM domain. As a result of these experiments, we propose a simple model of Gs-linked activation of CLR is to consider the receptor as being divided into distinct zones. Peptide agonist binding may predominantly be mediated by the ECLs. Changes in their conformation could be transmitted to key residues such as H295 and F349 at the extracellular ends of TMs 5 and 6. We suggest that these lead to repacking of TMs 3, 5 and 6 and a resetting of hydrophobic and hydrophilic networks. This then leads to the opening of the G protein-binding pocket on the cytosolic surface of the receptor, in a manner broadly compatible with that seen in class A GPCRs. The role of individual residues in the upper part of the TM-bundle is modulated by RAMPs and is also ligand-specific. While there is broad conservation of networks of amino acids throughout class B GPCRs, elements of these show distinct adaptions in individual GPCRs.

## Conflict of interest

The authors declare that they have no conflicts of interest with the contents of this article.

## Author contributions

MJW, CSW, MG, DRP and DLH conducted experiments. CAR, JCM and JS performed the computational modeling. MJW, DRP and DLH contributed receptor mutants to the study. MJW, DRP, ACC, CAR, JCM and DLH interpreted the experiments and wrote the paper.
