# Supplementary figures and images for: Receptor activity-modifying protein dependent and independent activation mechanisms in the coupling of calcitonin gene-related peptide and adrenomedullin receptors to Gs
Source: Biochem Pharmacol. 2017 Oct 15;142:96–110. doi: 10.1016/j.bcp.2017.07.005 (PMC5609567; doi:10.1016/j.bcp.2017.07.005)

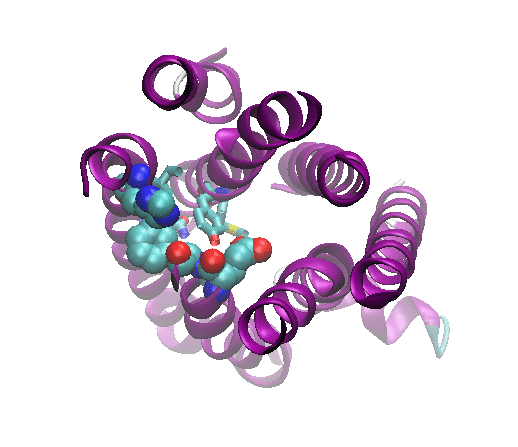

Supplement: Supplementary video 1 [file mmc1.gif]
